# Reporter immunogenicity as a defined rejection antigen: a noninvasive platform for skin transplant immunobiology

**DOI:** 10.3389/fimmu.2026.1859500

**Published:** 2026-09-01

**Authors:** Sydney Jeffs, John S. Wang, Reshma Goud, Jackie No, Gerald Lorio, Guang Han Lin, Nicole Moon, Violet Y. Tu, Timothy N. Trotter, Zachary C. Hartman, Tatiana Segura

**Affiliations:** 1 Medical Sciences Training Program, Duke University, Durham, NC, United States; 2 Department of Biomedical Engineering, Duke University, Durham, NC, United States; 3 Department of Surgery, Duke University, Durham, NC, United States

**Keywords:** Bioluminescence imaging, biomaterial scaffolds, immune tolerance, reporter immunogenicity, skin transplantation, transplant rejection

## Abstract

**Background:**

Preclinical transplantation models largely rely on macroscopic or terminal assessments of graft fate, limiting longitudinal evaluation of immune rejection and tolerance induction. Reporter proteins such as enhanced green fluorescent protein (eGFP) and firefly luciferase enable real-time, non-invasive imaging of transplanted tissues, but their utility in immunocompetent hosts is constrained by their immunogenicity as non-murine xenoantigens capable of eliciting robust adaptive immune responses.

**Methods:**

Full-thickness skin grafts from CAG-eGFP-luciferase donor mice were transplanted onto genetically tolerant (NoGlow^+^) or non-tolerant (NoGlow^-^) littermate recipients. Graft viability was monitored longitudinally by fluorescence and bioluminescence imaging. The impact of pharmacologic immunosuppression on anti-reporter immune responses and graft signal retention was evaluated in wild-type recipients. Multiplex transplantation experiments, in which multiple grafts were placed on individual animals, were performed to enable direct comparison of biomaterial-based interventions.

**Results:**

Reporter antigens served as defined, trackable targets of antigen-specific immune rejection in non-tolerant recipients, with progressive loss of graft signal correlating with rejection kinetics. Tolerant NoGlow^+^ recipients maintained stable reporter signal throughout the observation period. Pharmacologic immunosuppression mitigated anti-reporter immune responses and preserved bioluminescent signal in wild-type hosts. Multiplex transplantation within individual animals enabled side-by-side comparison of biomaterial therapies without invasive tissue sampling or early euthanasia.

**Conclusions:**

These findings establish a fluorescent and bioluminescent reporter-based skin graft platform that provides quantitative, non-invasive, longitudinal readouts of transplant viability and immune rejection. This system offers a flexible and tractable framework for studying transplant immunobiology and accelerating preclinical evaluation of immunomodulatory and biomaterial-based strategies aimed at improving graft outcomes.

## Introduction

Immune-mediated rejection remains a central barrier to durable graft survival in solid organ and tissue transplantation. While advances in immunosuppressive therapy have significantly improved short-term graft outcomes, long-term success continues to be limited by chronic rejection and the systemic toxicities associated with lifelong immunosuppression (1, 2). Progress towards more targeted immunomodulatory strategies is constrained, in part, by the limitations of existing preclinical models. The transplant field varies the degree of donor-recipient mismatch to model different clinical questions: fully MHC-mismatched combinations like C57BL/6 to BALB/c produce the rapid, stringent rejection used in many immunosuppression and mechanism studies (3, 4). MHC-matched but minor-antigen-mismatched grafts model the more clinically realistic scenario faced by matched cell and organ therapies. In both, the B- and T-cell response they provoke is polyclonal rather than directed at any single defined target, and as such it becomes difficult to isolate which antigen or pathway is driving a given rejection event, or to use graft outcome as a clean quantitative readout of a specific therapeutic intervention. Indeed, these model systems have relied primarily on macroscopic assessment of graft necrosis or terminal histologic analysis, providing limited temporal resolution and precluding longitudinal monitoring of graft viability (4, 5).

Reporter proteins such as enhanced green fluorescent protein (eGFP) and firefly luciferase (Luc) have enabled real-time tracking of cellular and tissue dynamics in oncology and regenerative medicine, and interest in applying these tools to transplantation research has grown (6–10). However, their utility in immunocompetent hosts is constrained by their immunogenicity as non-mammalian xenoantigens capable of driving robust adaptive immune responses. Our recent work addressed this limitation through development of the NoGlow mouse model, in which Cre-inducible expression of enzymatically inactive but antigenically intact variants of GFP and luciferase induces central and peripheral immune tolerance without producing detectable signal (11). This system enabled stable engraftment and longitudinal imaging of reporter-expressing tumors in tolerant recipients, while wild-type hosts rapidly rejected the same cells, demonstrating both the immunogenic potential of these reporters and feasibility of leveraging defined antigens to study antigen-specific immune responses *in vivo (*11).

Whether these principles extend to composite tissue transplantation biology, and whether reporter-based imaging can provide dynamic, quantitative readouts of graft fate and treatment response has not been investigated. In the present study, we applied the NoGlow tolerance system to murine skin transplantation to examine the immunologic consequences of reporter antigen expression in transplanted tissues, evaluate reported signal as a surrogate measure of graft viability, and demonstrate the utility of this platform for comparative assessment of immunosuppressive interventions. After establishing eGFP-luciferase as a trackable xenoantigenic readout of graft fate over time, we extended this approach to a multiplex transplantation platform, enabling direct, within-animal comparison of biomaterial-based therapies.

## Methods

### Animals and NoGlow mouse model

All animal procedures were performed in accordance with protocols approved by the Institutional Animal Care and Use Committee (IACUC) at Duke University under A001-24-01. CAG-GFP-luciferase (B6;FVB-*Ptprc^a^* Tg(CAG-luc,-GFP)L2G85Chco *Thy1^a^*/J) mice from the Jackson Laboratory (#025854) were used as skin graft donors in all experiments. Recipient mice included NoGlow^+^ (B6.Cg-Gt(ROSA)26Sor^tm1.2(CAG-rtTA3,-GFP*,-luc*)Zhrt^ Tyr^c-2J^/ZhrtJ) mice (Jackson Laboratories, #039259), NoGlow^-^ (littermate controls), and wild-type C57BL/6J mice (Jackson Laboratories, #000664). The NoGlow+ mouse model was generated as previously described (11). Briefly, NoGlow^+^ mice harbor a Cre-inducible cassette encoding enzymatically inactive variants of eGFP and firefly luciferase, whose developmental expression induces central and peripheral tolerance to the reporter antigens without producing detectable signal. NoGlow^-^ littermates lack Cre-mediated expression and remain immunologically naïve to both reporters. Mice were maintained under specific pathogen-free conditions and used at 8–16 weeks of age.

### Murine skin transplantation

Full-thickness dorsal skin grafts were harvested from CAG-eGFP-Luc donor mice. Donor skin was carefully debrided of panniculus carnosus and maintained on saline-moistened gauze on ice prior to transplantation (approximately 30 minutes cold ischemia; <20 minutes warm ischemia). Recipient mice (NoGlow^+^, NoGlow^-^ C57Bl/6) were anesthetized with 2% isoflurane and maintained on heated pads. Four equidistant full-thickness 8 mm defects or a single 1 × 1 cm dorsal defect were created, treatment was administered to the wound bed where applicable, and donor skin was sutured into place using 5–0 Prolene (Ethicon). Grafts were covered with Xeroform and Tegaderm and secured with a bolster dressing, which remained in place throughout the duration of the study to protect the transplanted grafts from mechanical injury. Bolster dressings were removed immediately prior to interval imaging studies and replaced before mice were returned to their housing. In multiplex experiments, four 8 mm grafts were placed on the dorsum of each recipient mouse, with each graft assigned to a distinct treatment condition. Graft survival was assessed longitudinally by macroscopic inspection and imaging, with investigators blinded to treatment conditions where applicable.

### Pharmacologic immunosuppression

For immunosuppression studies, recipient NoGlow- C57BL/6 mice received intraperitoneal injections of CTLA4-Ig (Belatacept) and rapamycin (12). Rapamycin (2 mg/kg) was administered every third day beginning one day prior to transplantation, for a total of 6 doses. Belatacept (10 mg/kg) was administered twice weekly starting on the day of transplantation, for a total of 4 doses.

### *In vivo* imaging

Graft viability was monitored by fluorescence (GFP) and bioluminescence (luciferase) imaging on an IVIS system. For bioluminescence, mice received 50 µL of 30 mg/kg D-luciferin intraperitoneally 10 minutes prior to imaging (1-minute exposure, medium binning, f/1). GFP fluorescence was acquired at 465 nm excitation/520 nm emission with identical settings across all time points. Images were analyzed in Living Image (v4.3, PerkinElmer) with fixed ROIs over each graft site. Signal was quantified as average radiance [p/s/cm²/sr] for bioluminescence and average radiant efficiency ([p/s/cm²/sr]/[µW/cm²]) for fluorescence.

### Microfluidic device fabrication

Water-in-oil droplet microfluidic devices were fabricated using standard soft lithography techniques. Briefly, polydimethylsiloxane (PDMS; Sylgard™ 184, Dow) base and crosslinker were mixed at a 10:1 ratio, poured over silicon wafer masters, degassed, and cured at 60 °C overnight. Devices were sealed to glass slides via plasma treatment (Tesla coil, 2 min, maximum voltage), vacuum dried, and incubated overnight at 60 °C. Prior to use, channels were rendered hydrophobic by flushing with polysiloxane (Rain-X) (13).

### HA-norbornene microgel synthesis and MAP scaffold formation

HA-NB microgels were synthesized via thiol-ene click chemistry within microfluidic droplet generators as previously described by our group (14, 15). Microgels were annealed into MAP scaffolds via tetrazine-norbornene click chemistry; scaffold stiffness was tuned by the HA-tetrazine to HA-NB molar ratio (Tet/NB = 7 for *in vivo* studies).

### Rheological characterization and microgel sizing

Mechanical properties of Woun’Dres, bulk hydrogels, and MAP scaffolds were assessed using rotational rheometry (Anton Paar) with parallel plate geometry. Frequency sweeps (0.1–100 rad s^-^¹) were performed at 3% strain, and storage (G′) and loss (G″) moduli were recorded. Microgel size distributions were quantified from fluorescence microscopy images (Nikon Ti-Eclipse) using custom MATLAB scripts. Mean particle diameters and standard deviations were calculated from multiple fields of view.

### Biomaterial treatment groups

**Figure 1:**
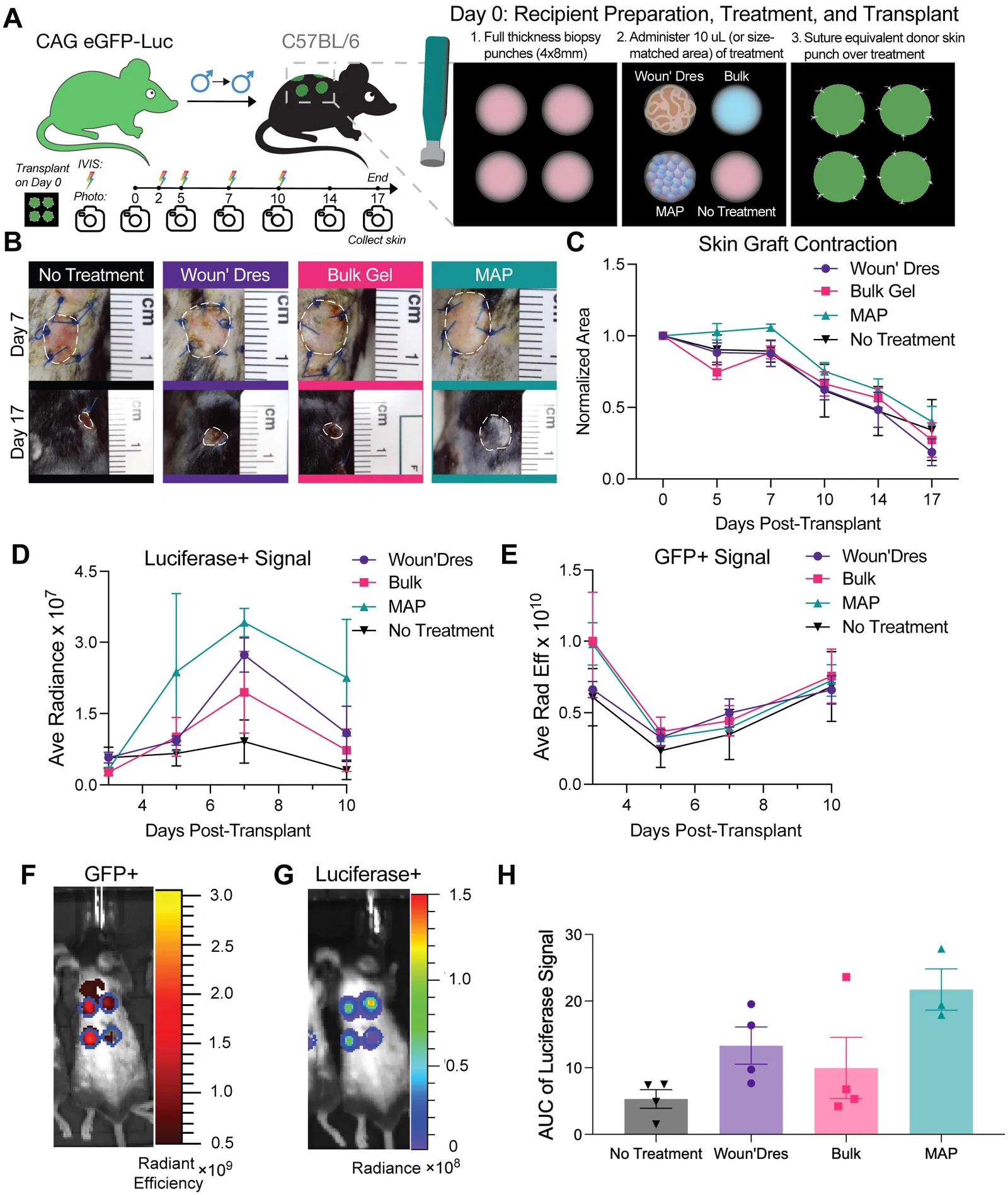
Multiplex fluorescent grafts enable rapid screening of biomaterial therapies in a multiplex transplant model. **(A)** Experimental schema for multiplex skin graft transplantation. Four 8-mm full-thickness skin grafts from CAG-eGFP-Luc donors were transplanted onto each NoGlow- C57BL/6 recipient. **(B)** Representative images of grafts following treatment at days 7 and 17 post-transplant. **(C)** Quantification of wound contraction. **(D, E)** Longitudinal analysis of reporter signal by luciferase bioluminescence **(D)** and GFP fluorescence **(E)**. **(F, G)** Representative IVIS images of multiplex grafts at day 7. **(H)** Luciferase signal (total flux, photons/sec) was quantified by area under the curve (AUC), calculated using GraphPad Prism with the trapezoidal rule, a baseline of Y = 0, and integration across the full imaging time course (Days 2–10). Statistical analysis for panel H was performed using a mixed-effects model (REML) to account for repeated measures within each mouse (four graft conditions per animal), followed by Holm-Sidak’s multiple comparisons test with a single pooled variance. Data are presented as mean ± SEM.

**Figure 2:**
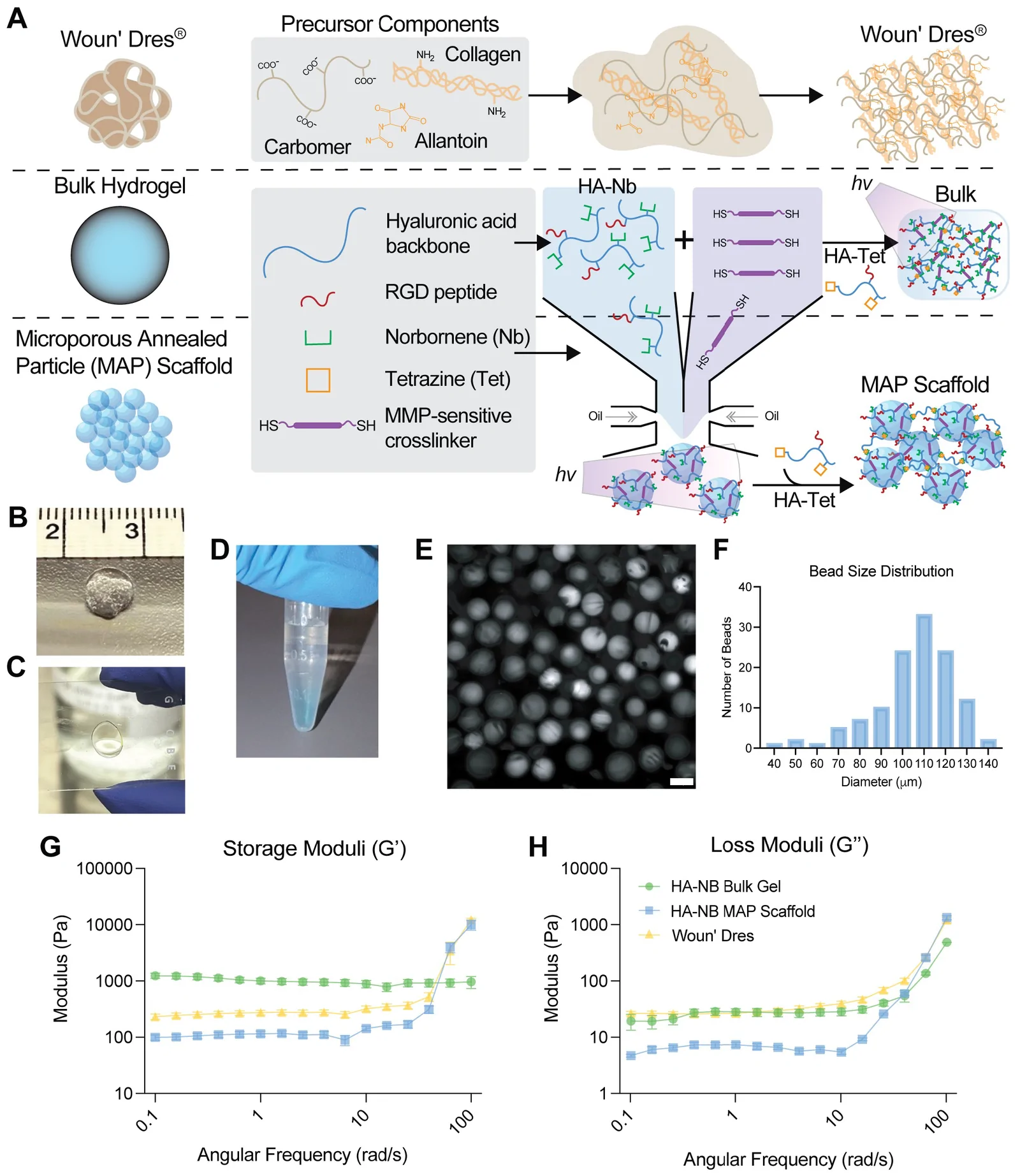
Characterization of biomaterial platforms for localized modulation of skin graft healing. **(A)** Biomaterial design and composition. **(B–D)** Representative images of biomaterials. Macroscopic images of Woun’Dres, cured bulk hydrogel, and MAP hydrogel microparticles prior to injection and annealing. **(E)** Confocal microscopy of hydrogel microparticles. Scale bar = 100 µm. Fluorescently labeled hydrogel particles demonstrate the spherical morphology used to generate MAP scaffolds. **(F)** Particle size distribution. Analysis shows monodisperse microparticles with an average diameter centered around ~110 µm. **(G)** Storage modulus (G′) frequency sweep. **(H)** Loss modulus (G″) frequency sweep.

For multiplex transplantation experiments, four 8 mm full-thickness dorsal defects were created in each recipient mouse, and skin grafts from CAG-eGFP-Luc donors were randomly assigned to one of four treatment conditions applied directly to the graft bed immediately before transplantation: (1) no treatment (untreated control); (2) Woun’Dres, purchased from manufacturer and applied using a positive-displacement pipette; (3) bulk HA-NB hydrogel, pre-crosslinked via thiol-ene chemistry and applied as an 8 mm diameter disc; or (4) MAP scaffold, delivered as a 10 µL suspension of HA-NB microgels using a positive-displacement pipette. Liquid formulations were applied to uniformly cover the wound bed, while bulk hydrogels were size matched to the defect. MAP scaffolds were allowed to anneal *in situ* for 30 minutes prior to graft placement. A schematic summarizing this process is depicted in **Figure 1A**. in Donor skin graft preparation, bolster dressing, and imaging protocols were performed as described above. Material composition, mechanical properties, and physical characterization are described in **Figure 2**. One graft assigned to the MAP scaffold group was excluded because of scaffold delivery failure prior to postoperative assessment and was excluded from all analyses.

### Tissue processing and flow cytometry

To facilitate lymph node identification, mice received intradermal injection of isosulfan blue immediately prior to sacrifice. Spleens and lymph nodes were harvested and processed into single-cell suspensions via mechanical dissociation and filtration through 70 µm strainers. Red blood cells were lysed using ACK buffer. Cells were first incubated with ViaDye Red and FcX blocker for 10 minutes and then stained with the following antibody cocktail for 30 minutes on ice: CD45 APC-Fire 810 (30-F11), CD19 BV421 (6D5), CD3 RB705 (17A2), CD8 Super Bright 645 (OKT8), CD4 FITC (GK1.5), CD62L Alexa Fluor 647 (MEL-14), and CD44 PE-Cy7 (IM7). Samples were then analyzed on a spectral flow cytometer (Cytek Northern Lights-CLC). Cells were first gated on forward and side scatter to exclude debris, followed by singlet discrimination using SSC-A versus SSC-H. Live immune cells were identified as CD45+ ViaDye Red-negative events. Within this population, CD19+ and CD3+ cells were gated separately, with CD19+ cells quantified directly. CD3+ cells were further subdivided into CD4+ and CD8+ populations, and fluorescence-minus-one (FMO) controls were used to define gates for CD44 and CD62L expression.

### Histology and immunohistochemistry

Skin grafts were harvested and bisected. One portion was embedded in OCT and snap-frozen in isopentane, while the other was fixed in 4% paraformaldehyde, paraffin-embedded, sectioned, and stained with hematoxylin and eosin (H&E). Sectioned skin grafts were additionally stained with anti-mouse CD8 (Cell Signaling Technology, D4W2Z, 1:500) or CD4 (Cell Signaling Technology, D7D2Z, 1:500) antibodies with hematoxylin counterstaining. The number of CD8+ or CD4+ T cells per 20x field, averaged across five fields per replicate, was quantified through Fiji/ImageJ.

### Xenoantigen-specific antibody assay

Serum samples were collected longitudinally via submandibular bleeding. Antibody responses against GFP and luciferase were measured by enzyme-linked immunosorbent assay (ELISA) as previously described (11). Briefly, plates were coated with recombinant GFP or luciferase protein and incubated with serially diluted serum samples. Bound antibodies were detected using horseradish peroxidase (HRP)-conjugated antibodies against total IgG (Cell Signaling Technology) or IgG subclasses (SouthernBiotech: IgG1 1070–05, IgG2a 1081–05, IgG2b 1091–05, IgG2c 1078–05), developed with 3,3’,5,5’-tetramethylbenzidine (TMB), and read at 450 nm. Comparative ELISAs used samples collected on the same day.

### Statistical analysis

Data are presented as mean ± standard error mean unless otherwise indicated. Statistical comparisons were performed using GraphPad Prism. Group comparisons were conducted using unpaired t-tests, one-way ANOVA, or area under curve with *post hoc* testing, as appropriate. Area under the curve was calculated using GraphPad Prism with the trapezoidal rule, a baseline of Y = 0, and integration across the full imaging time course (Days 2–10). A p-value <0.05 was considered statistically significant.

## Results

### Reporter-expressing grafts reveal and enable longitudinal quantification of antigen-specific rejection

**Figure 3:**
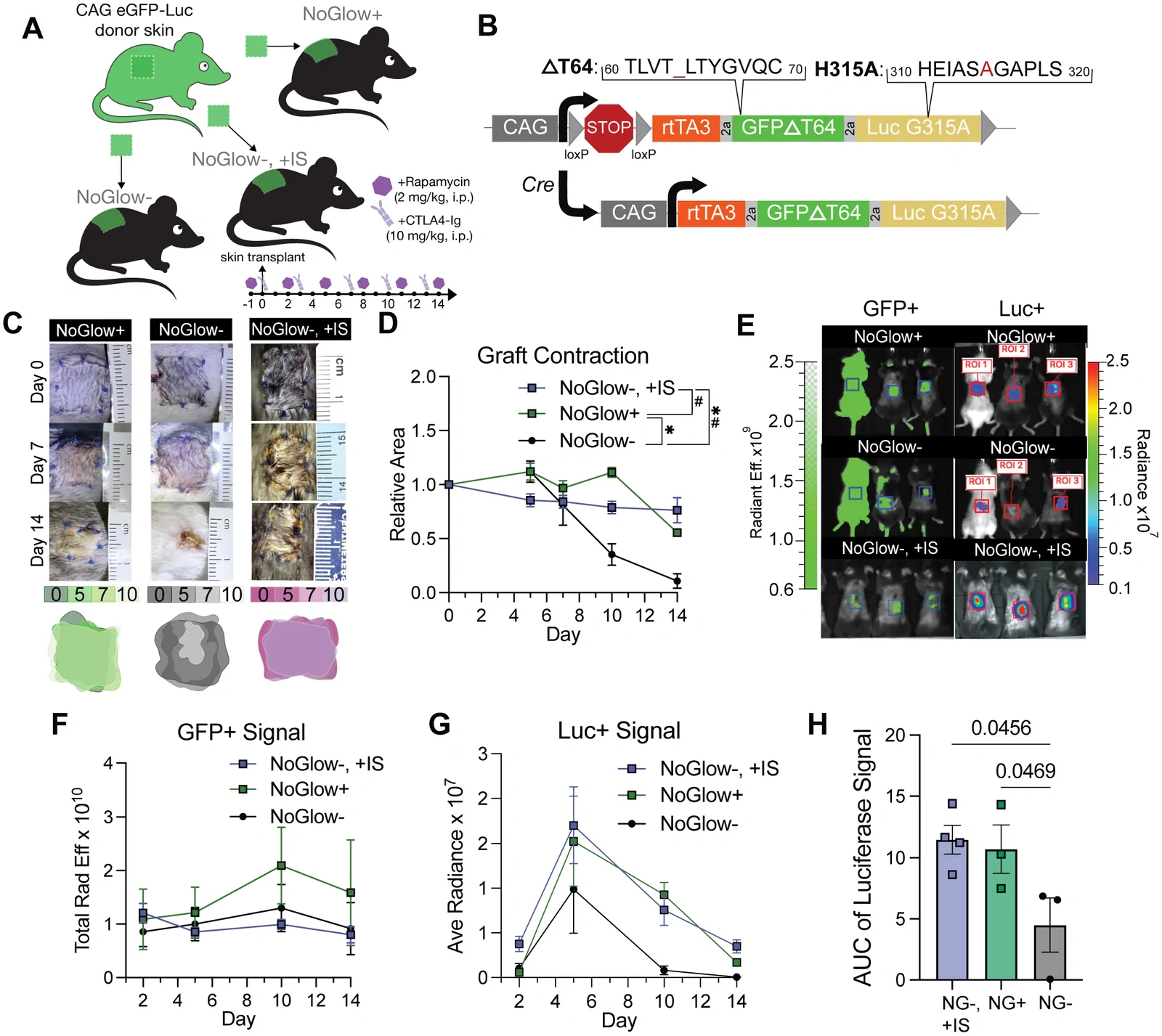
Reporter-expressing skin grafts enable longitudinal imaging of antigen-specific rejection and its attenuation by immunosuppression. **(A)** Experimental schema of fluorescent skin transplantation. Full-thickness skin from CAG-eGFP-Luc donor mice was transplanted onto sex-matched NoGlow^+^ (tolerant) or immunosuppressed or non-immunosuppressed NoGlow^-^ (non-tolerant) littermate recipients. **(B)** Genetic design of the NoGlow tolerance system. **(C)** Representative images of transplanted skin grafts with temporal overlay of graft boundaries across time. **(D)** Measurement of graft contraction over time. **(E)** Representative IVIS fluorescence and bioluminescence images at day 10 post-transplant. **(F)** Longitudinal GFP fluorescence imaging. **(G)** Longitudinal luciferase bioluminescent quantification. **(H)** Quantification of luciferase signal by AUC. Data are presented as mean ± SEM. n = 3 mice per group for NoGlow^+^ and non-immunosuppressed NoGlow^-^ controls; n = 4 mice for the immunosuppressed NoGlow^-^ group. Statistical analysis for panel H was performed using one-way ANOVA with Tukey’s multiple comparisons test.

To determine whether eGFP and Luciferase could function as tractable rejection antigens in a transplant model while enabling non-invasive monitoring of graft viability, we transplanted full-thickness skin from CAG-eGFP-luciferase donor mice onto sex-matched NoGlow^+^ or NoGlow^-^ littermate recipients (**Figure 3A**). In parallel, we evaluated a clinically relevant immunosuppressive regimen consisting of abatacept, a co-stimulation blocker and murine equivalent of belatacept, and rapamycin, an mTOR inhibitor, to benchmark rejection responses against non-tolerant (NoGlow^-^) and tolerant (NoGlow^+^) controls. In the NoGlow system, Cre-mediated recombination induces expression of enzymatically inactive but antigenically intact variants of GFP and luciferase, conferring immune tolerance to these reporters without generating detectable fluorescence or bioluminescence signal (**Figure 3B**).

Grafts were monitored longitudinally by macroscopic inspection and IVIS imaging. By day 14 post-transplant, NoGlow^+^ and immunosuppressed recipients maintained viable grafts with preserved tissue architecture, whereas NoGlow^-^ grafts exhibited marked contraction, necrosis, and crusting consistent with rejection (**Supplementary Figures 1A, B**). Temporal overlay of graft boundaries illustrated progressive reduction in graft area in non-tolerant recipients (**Figure 3C**) (16). Quantitative measurement of graft area confirmed progressive contraction and loss of viable graft tissue in NoGlow^-^ recipients relative to tolerant controls (**Figure 3D**).

Longitudinal IVIS imaging demonstrated divergence in reporter signal between groups that paralleled macroscopic outcomes (**Figure 3E**; **Supplementary Figures 1C, D**). GFP fluorescence was detectable in all groups early after transplantation but was more consistently maintained in NoGlow^+^ and immunosuppressed recipients, although this difference did not reach statistical significance (**Figure 3F**). In contrast, luciferase bioluminescence imaging revealed a clear separation between groups, with signal better preserved in NoGlow^+^ and immunosuppressed mice and progressively declining in NoGlow^-^ animals over the post-transplant period (**Figures 3G, H**). Quantification of total flux by area under the curve demonstrated a significant difference across the 14-day study period.

**Figure 4:**
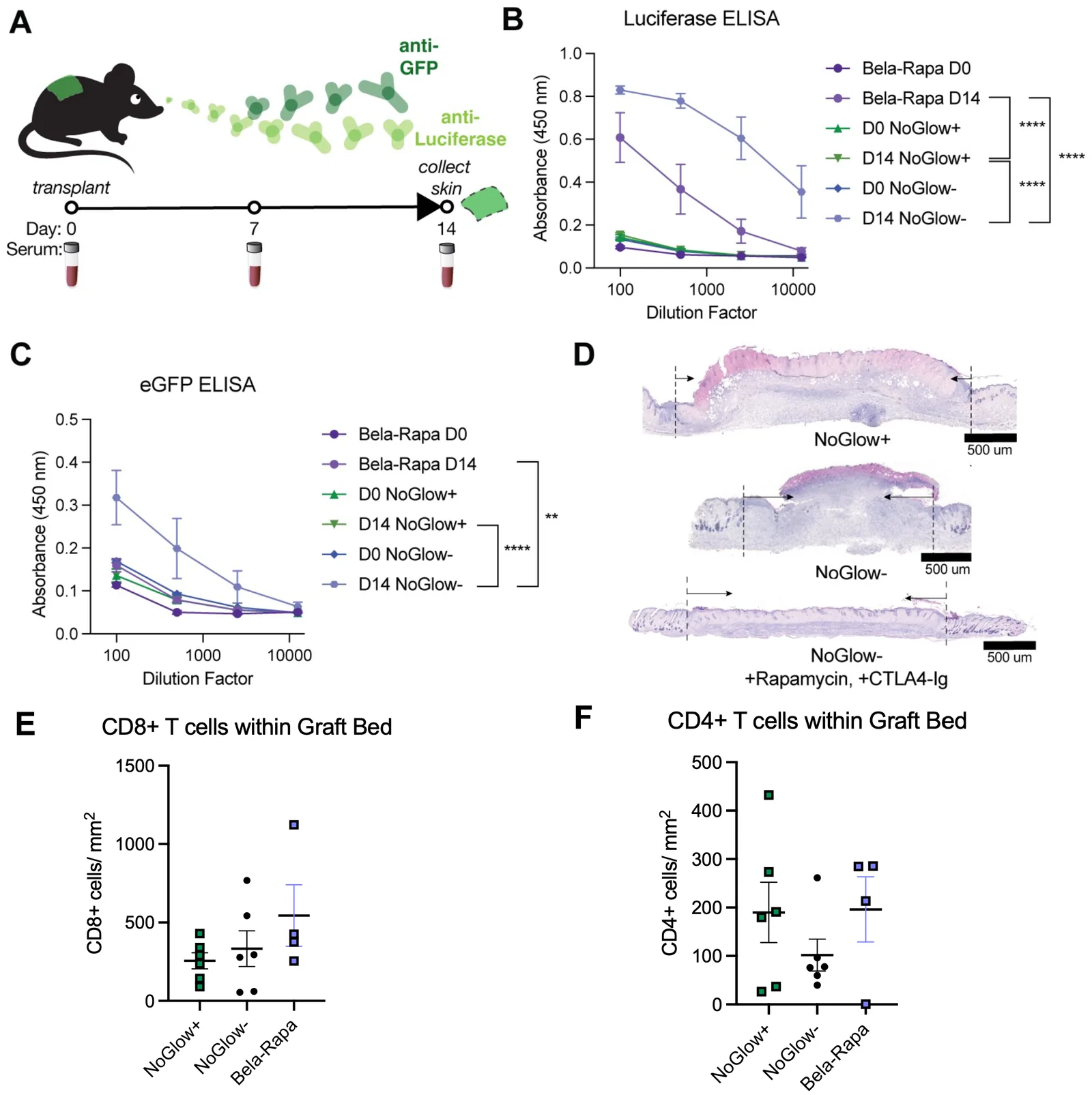
Reporter-specific humoral responses correlate with graft rejection and are modulated by immunosuppression. **(A)** Experimental design for evaluation of anti-reporter antibody responses following skin transplantation. **(B)** Anti-luciferase antibody as measured by ELISA. **(C)** Anti-GFP antibodies as measured by ELISA. **(D)** Representative hematoxylin and eosin-stained sections of skin grafts from NoGlow+, NoGlow- control, and NoGlow- immunosuppressed recipients at endpoint. **(E, F)** IHC quantification of graft-infiltrating CD8+ and CD4+ T cells. Data are presented as mean ± SEM. ELISA data **(B, C)** at 1:100 dilution and IHC data **(E, F)** were analyzed using one-way ANOVA with Holm-Sidak’s multiple comparisons test. Dashed lines in **(D)** denote graft boundaries, and arrows indicate the extent of the graft bed. **(E)** Scale bar, 500 μm. **P < 0.01, ****P <.0001.

To determine whether reporter-specific humoral immunity contributed to graft rejection in non-tolerant hosts, we measured serum antibodies against GFP and luciferase following transplantation (**Figure 4A**). ELISA assessment revealed that both NoGlow^+^ and immunosuppressed mice were significantly less capable of eliciting eGFP and Luciferase graft antigen-specific antibody responses compared with NoGlow^-^ recipients (**Figures 4B, C**). Notably, while anti-Luciferase IgG antibodies were significantly lower in the immunosuppressed mice compared to the non-immune suppressed mice, these responses were still significantly elevated in comparison to those observed in NoGlow+ mice. These results support the findings that pharmacologic immunosuppression attenuates, but does not fully abrogate, antigen-specific humoral responses, in line with previous studies (17–19).

To further characterize the isotype composition of the anti-Luciferase humoral response, we performed subclass-specific ELISAs for IgG1, IgG2b, IgG2c, and IgG3 (**Supplementary Figures 2A–D**). The increase in anti-Luciferase titers observed in NoGlow^-^ recipients between day 0 and day 14 was maintained across all four subclasses, and this increase was significantly attenuated across all subclasses in NoGlow^+^ mice over the same interval. In contrast, immunosuppressed mice showed a significant increase in IgG1 and IgG2b titers between day 0 and day 14, while IgG2c and IgG3 titers did not differ significantly between these timepoints, suggesting that Belatacept-rapamycin treatment prevents class switching to IgG2c and IgG3, but not to IgG1 or IgG2b, by this timepoint.

To validate our bioluminescent measurement as a proxy for graft rejection, we also performed histologic evaluation of transplanted skin at endpoint. These assessments revealed distinct tissue-level differences between groups, characterized by graft contraction and architectural remodeling (**Figure 4D**; **Supplementary Figures 2E–G**). In NoGlow^+^ and immunosuppressed grafts, the epidermis remained largely intact with preserved continuity, and the dermis exhibited organized collagen deposition with minimal inflammatory infiltrate, consistent with maintained tissue integrity. In contrast, NoGlow^-^ grafts displayed features of tissue injury, including epidermal attenuation, dermal compaction, and increased cellularity consistent with inflammatory infiltration.

We next assessed CD4+ and CD8+ T cell infiltration in the localized skin graft bed (**Figures 4E, F**; **Supplementary Figure 2H**), but observed no difference among the NoGlow+, NoGlow−, and Bela/Rapa NoGlow− groups. We additionally performed bulk immune profiling of peripheral lymphoid compartments in NoGlow+ and untreated NoGlow- mice at the day 14 endpoint to corroborate localized skin findings (**Supplementary Figure 2I**). No significant differences were observed in the frequency of major lymphocyte populations, including CD4^+^ and CD8^+^ T cells and CD19^+^ B cells in splenocytes or draining lymph nodes. Phenotypic analysis of CD4^+^ and CD8^+^ T cells similarly showed no differences in effector memory (CD44^+^CD62L^-^), naïve (CD44^-^CD62L^+^), or central memory (CD44^+^CD62L^+^) subsets. Together, these results establish that reporter-driven rejection can be robustly quantified through histologic, serologic, and longitudinal imaging readouts, even in the absence of overt changes in localized or systemic lymphocyte composition.

### Dual reporter grafts enable comparative evaluation of biomaterial-based therapies

To determine whether reporter-based imaging could enable within-animal comparison of localized therapeutic interventions, we transplanted four 8-mm full-thickness skin grafts from CAG-eGFP-Luc donors onto each NoGlow- C57Bl/6 recipient. This multiplex design enabled direct comparison of treatment effects across spatially distinct grafts within a shared host environment. Each graft site received a distinct treatment: no treatment, Woun’Dres, bulk HA-NB hydrogel, or MAP scaffold (**Figure 2A**). Materials were selected to represent one clinically used material and two hyaluronic acid-based materials with different matrix architectures and physical properties (14, 20).

Prior to transplantation, the physical and mechanical properties of all three materials were characterized (**Figures 2B–D**). Confocal microscopy of fluorescently labeled microparticles confirmed the spherical morphology of the MAP scaffold building blocks (**Figure 2E**), and particle size analysis revealed a monodisperse population with a mean diameter of approximately 110 μm (**Figure 2F**). Rheological characterization showed that bulk HA-NB hydrogels exhibited substantially higher storage moduli than either Woun’Dres or MAP scaffolds, which both displayed lower stiffness consistent with more compliant, tissue-like behavior (**Figure 2G**). Loss modulus measurements further distinguished the materials: MAP scaffolds exhibited frequency-dependent viscoelastic behavior characteristic of particulate networks, reflecting dynamic interparticle interactions, whereas bulk hydrogels displayed comparatively stable mechanical responses across the tested frequency range (**Figure 2H**) (21, 22).

For this experimental design (**Figures 1A, B**), we observed that during the peak window, MAP-treated grafts tended to exhibit the lowest level of skin contraction (**Figures 1B, C**; **Supplementary Figure 3A**), as well as significantly higher luciferase signal intensity (compared to controls), while untreated grafts trended lowest (**Figure 1D**; **Supplementary Figure 4B**). In contrast, GFP fluorescence showed minimal separation between groups over time (**Figure 1E**; **Supplementary Figure 4C**), again indicating reduced sensitivity for detecting treatment effects. Representative IVIS imaging of multiplex grafts at day 7 are shown in **Figures 1F, G**. Quantification by area under the curve showed a trend toward increased luciferase signal in MAP-treated grafts relative to untreated controls, although this difference did not reach statistical significance after accounting for the non-independence of graft conditions within each mouse (**Figure 1H**). Together, these findings suggest that dual-reporter skin grafts enable within-animal, spatially resolved comparison of localized biomaterial therapies, with luciferase-based imaging providing a more sensitive readout of early, transient differences in graft viability than GFP fluorescence or gross morphologic assessment, though larger cohorts will be needed to confirm treatment-specific effects.

### Discussion

The present study demonstrates that fluorescent and bioluminescent reporter proteins, long recognized as immunogenic in oncology models, elicit antigen-specific rejection when expressed in transplanted skin and can be leveraged to create a tractable, quantifiable model of transplant immunobiology. Accordingly, NoGlow^+^ recipients maintained graft-associated bioluminescent signal, whereas NoGlow^-^ recipients exhibited progressive signal loss consistent with rejection. This reframing of reporter immunogenicity from a technical limitation into an experimental asset has important implications for the design and evaluation of preclinical transplant models.

A key distinction between this system and conventional allograft models is the use of a defined antigen. Standard murine transplantation involves complex immune responses directed against highly polymorphic MHC antigens and numerous minor histocompatibility disparities, making it difficult to attribute rejection to specific antigenic targets or to quantify therapeutic responses (23). In contrast, the expression of these reporter proteins in an MHC identical background provide a controlled antigen system in which the target of immune rejection is known, its expression can be genetically specified, and the immune response can be directly measured by serology and imaging. This reductionist approach enables more precise interrogation of antigen-specific responses and immunomodulatory interventions, albeit without fully recapitulating the complexity of clinical transplant immunology.

In our studies, clinically validated immunosuppressive agents combining co-stimulation blockade and mTOR inhibition attenuated anti-reporter antibody formation and preserved graft-associated bioluminescent signal in wild-type recipients. These findings support the biological relevance of reporter antigen-driven rejection as a model of antigen-specific immune responses and suggest that this platform may be useful for evaluating immunosuppressive strategies. The concordance between reduced antibody formation and retention of reporter signal further supports the use of imaging-based endpoints as pharmacodynamic readouts of therapeutic activity.

The absence of significant differences in localized T-cell frequencies or bulk peripheral lymphocyte frequencies and memory phenotype distribution, despite clear evidence of reporter-driven rejection by histology, serology, and imaging, suggests that a single endpoint timepoint may not reliably capture the underlying immune response. Because histologic rejection becomes apparent at a different timepoint across animals, the immune response driving rejection may have already resolved in some mice by the time these changes are detected. Alternatively, total CD4+ and CD8+ T cell numbers may not differ between tolerant and non-tolerant mice, while the proportion of functional T cells within these populations could differ, a distinction that bulk profiling does not capture. Further studies, including T cell analysis for regulatory and effector populations or sampling at additional timepoints, are warranted to resolve these possibilities. From a practical standpoint, this dissociation may suggest that histologic, serologic, and imaging-based readouts rather than peripheral immunophenotyping are more sensitive and reliable means of quantifying reporter-driven rejection in this system.

An additional advantage of this system is the ability to perform multiplex transplantation experiments within individual animals. By implanting multiple reporter-expressing grafts in the same recipient, we were able to directly compare the effects of different biomaterial treatments under identical systemic immune conditions. This approach reduces inter-animal variability and allows for more efficient evaluation of localized therapeutic strategies. In the present study, we applied this framework to examine the effects of hydrogel-based biomaterials designed to modulate the wound environment. MAP scaffolds showed a trend toward greater signal retention at early time points relative to bulk hydrogel and collagen dressing conditions; however, this difference did not reach statistical significance, which may reflect the limited statistical power of our modest cohort size (24, 25). These findings are consistent with prior reports that the interconnected porosity of MAP networks facilitates cell infiltration and vascular ingrowth, although the durability and mechanistic basis of these effects require further investigation.

Within our multiplex study, we observed a transient rise in luciferase signal observed across all treatment groups, including untreated controls, at approximately day 7 post-transplant. This may reflect progressive graft revascularization rather than an increase in cell viability per se. Because D-luciferin substrate delivery to the graft depends on local blood flow, early post-transplant hypoperfusion could transiently limit substrate availability, with subsequent revascularization improving delivery independent of changes in viable cell number. This possibility should be considered when interpreting luciferase signal as a surrogate for graft viability during the early post-transplant period. Direct assessment of vascular density (e.g., CD31 staining) at this timepoint would help distinguish these possibilities; however, in the current study tissue was collected only at the day 14 endpoint, by which time all grafts had undergone rejection, precluding meaningful assessment of vascularization at the time of peak luciferase signal. Future studies incorporating interim tissue collection at earlier timepoints will be needed to directly test this hypothesis. One final limitation of the biomaterial comparison experiment is that it was performed exclusively in non-tolerant NoGlow^-^ recipients undergoing active rejection throughout the observation period. As such, differences in luciferase signal between biomaterial conditions likely reflect a combination of immune rejection dynamics and biomaterial-intrinsic effects on graft vascularization and viability that cannot be fully separated in the current experimental design. Future studies incorporating a NoGlow^+^ tolerant cohort treated with the same biomaterial conditions would help isolate biomaterial-specific effects on graft outcome independent of alloimmune rejection.

Despite these limitations, the fluorescent graft platform provides several advantages for experimental transplantation research. Longitudinal, non-invasive monitoring of graft fate enables tracking of rejection dynamics over time without the need for repeated biopsies or terminal endpoints, while defined reporter antigens further provide a controlled system for studying antigen-specific immune responses, evaluation of local immunomodulatory strategies, and screening of novel immunosuppressive agents, offering improved temporal resolution and experimental efficiency relative to conventional models.

## Data Availability

The original contributions presented in the study are included in the article/**Supplementary Material**. Further inquiries can be directed to the corresponding author.
