# Supplementary material for: Reporter immunogenicity as a defined rejection antigen: a noninvasive platform for skin transplant immunobiology
Source: Front Immunol. 2026 Sep 1;17:1859500. doi: 10.3389/fimmu.2026.1859500 (PMC13574563; doi:10.3389/fimmu.2026.1859500)
Supplement: Supplementary file 1 [file DataSheet1.pdf]

Supplementary Information

Reporter Immunogenicity as a Defined Rejection Antigen: A Noninvasive Platform for Skin Transplant Immunobiology

Sydney Jeffs[1,2†], John S. Wang[1,3†], Reshma Goud[2], Jackie No[2], Gerald Lorio[2], Guang Han Lin[3], Nicole Moon[3], Violet Y. Tu[1], Timothy N. Trotter[3], Zachary C. Hartman[3], and Tatiana Segura[2]*

[1] Medical Sciences Training Program, Duke University, Durham, NC, USA

[2] Department of Biomedical Engineering, Duke University, Durham, NC, USA

[3] Department of Surgery, Duke University, Durham, NC, USA

*corresponding author. Email: tatiana.segura@duke.edu

[†] These authors contributed equally to this work.

*corresponding author.

Supplementary Information includes:

Supplementary Figures S1 to S3.

Supplementary Figure 1.

A

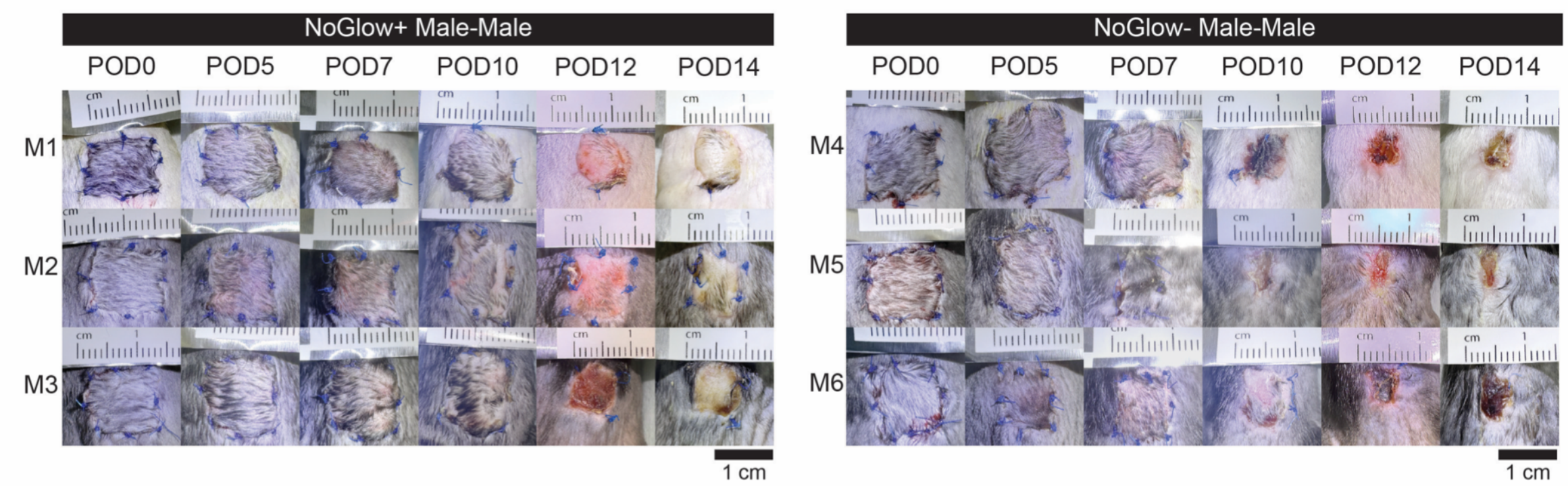

B

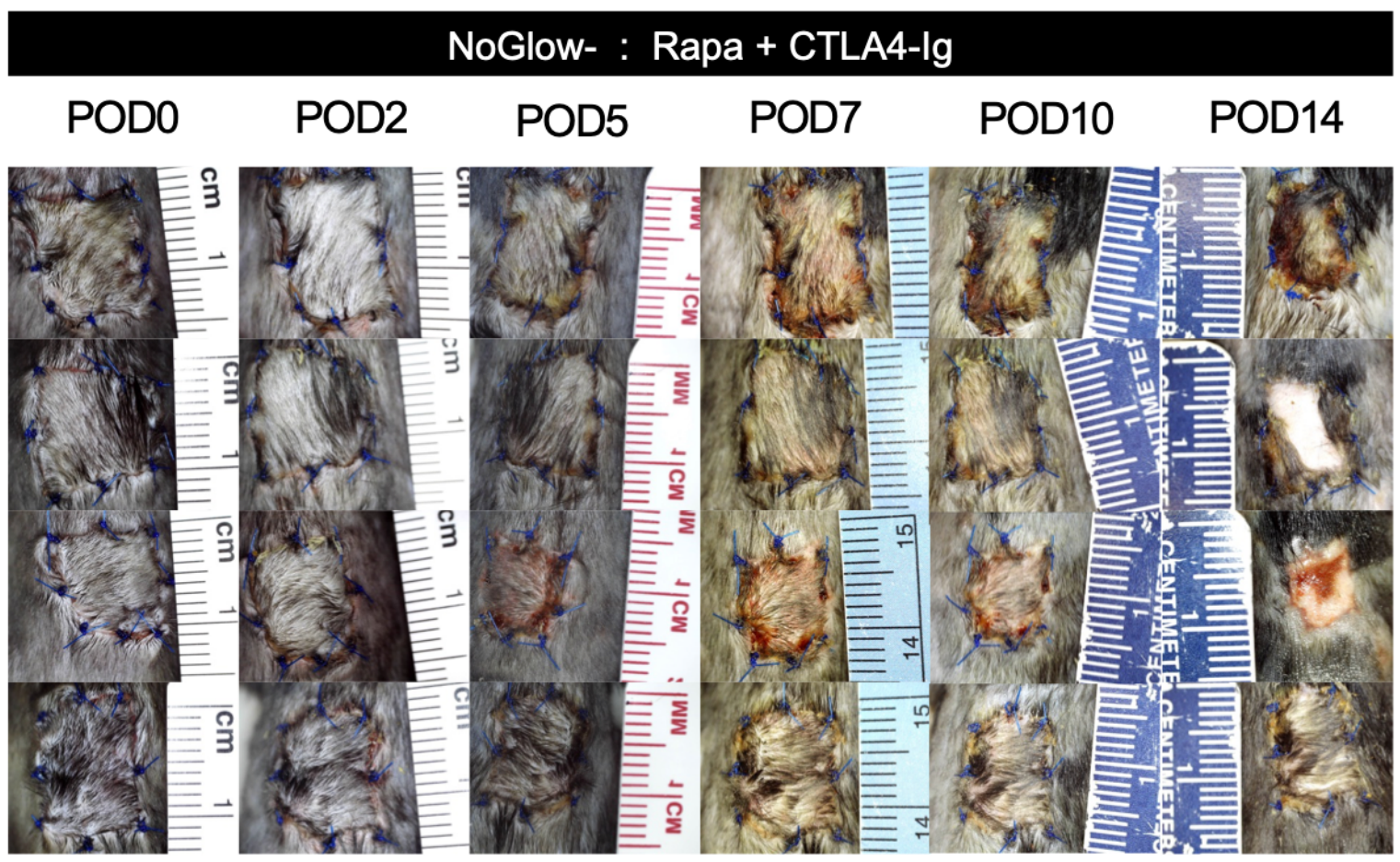

C

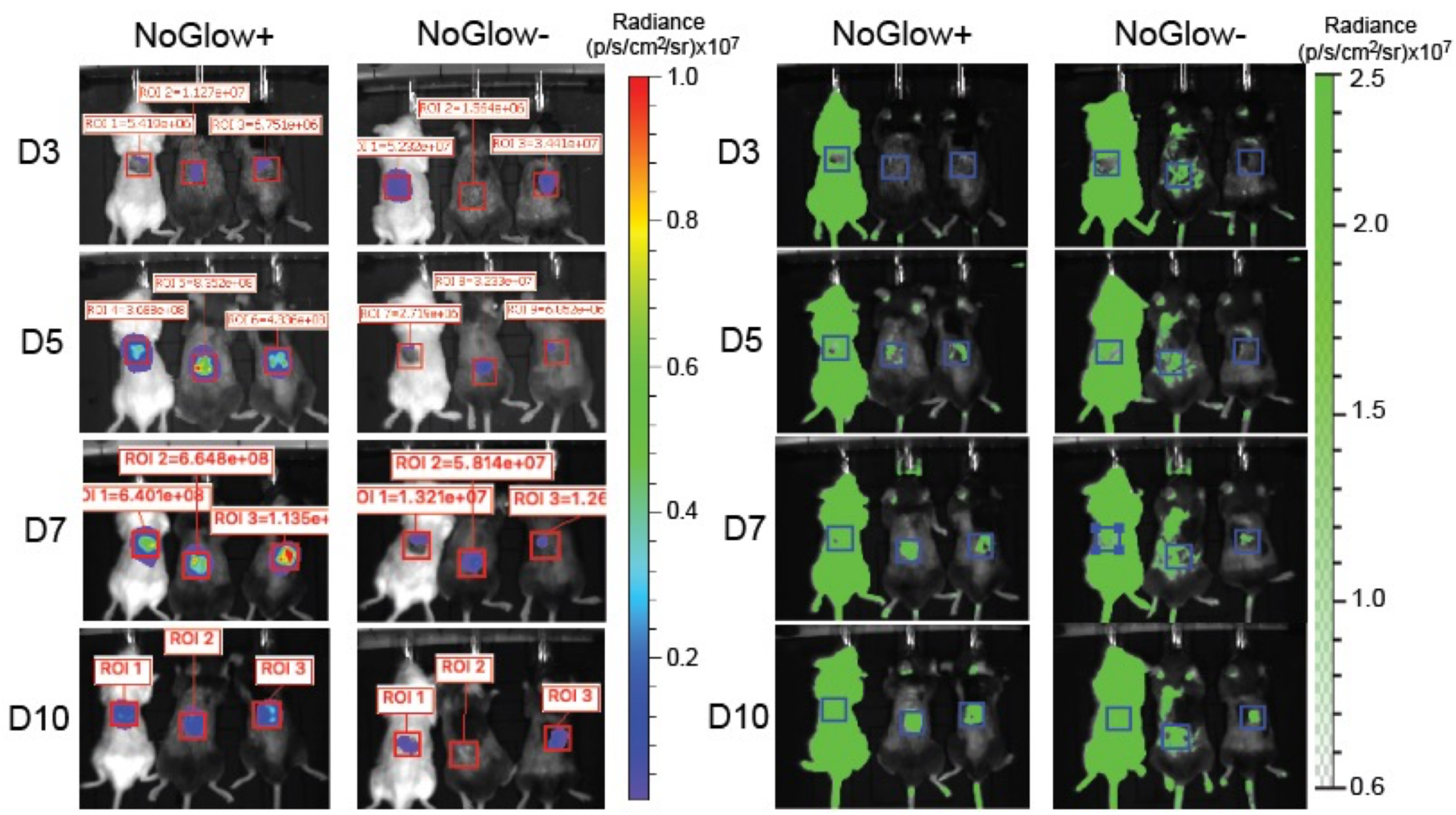
NoGlow+
NoGlow-
Radiance (p/s/cm2/sr)x10^7
1.0
0.8
0.6
0.4
0.2
NoGlow+
NoGlow-
Radiance (p/s/cm2/sr)x10^7
2.5
2.0
1.5
1.0
0.6
D3
D5
D7
D10
ROI 2=6.648e+08
ROI 2=5.814e+07
ROI 1
ROI 2
ROI 3

D

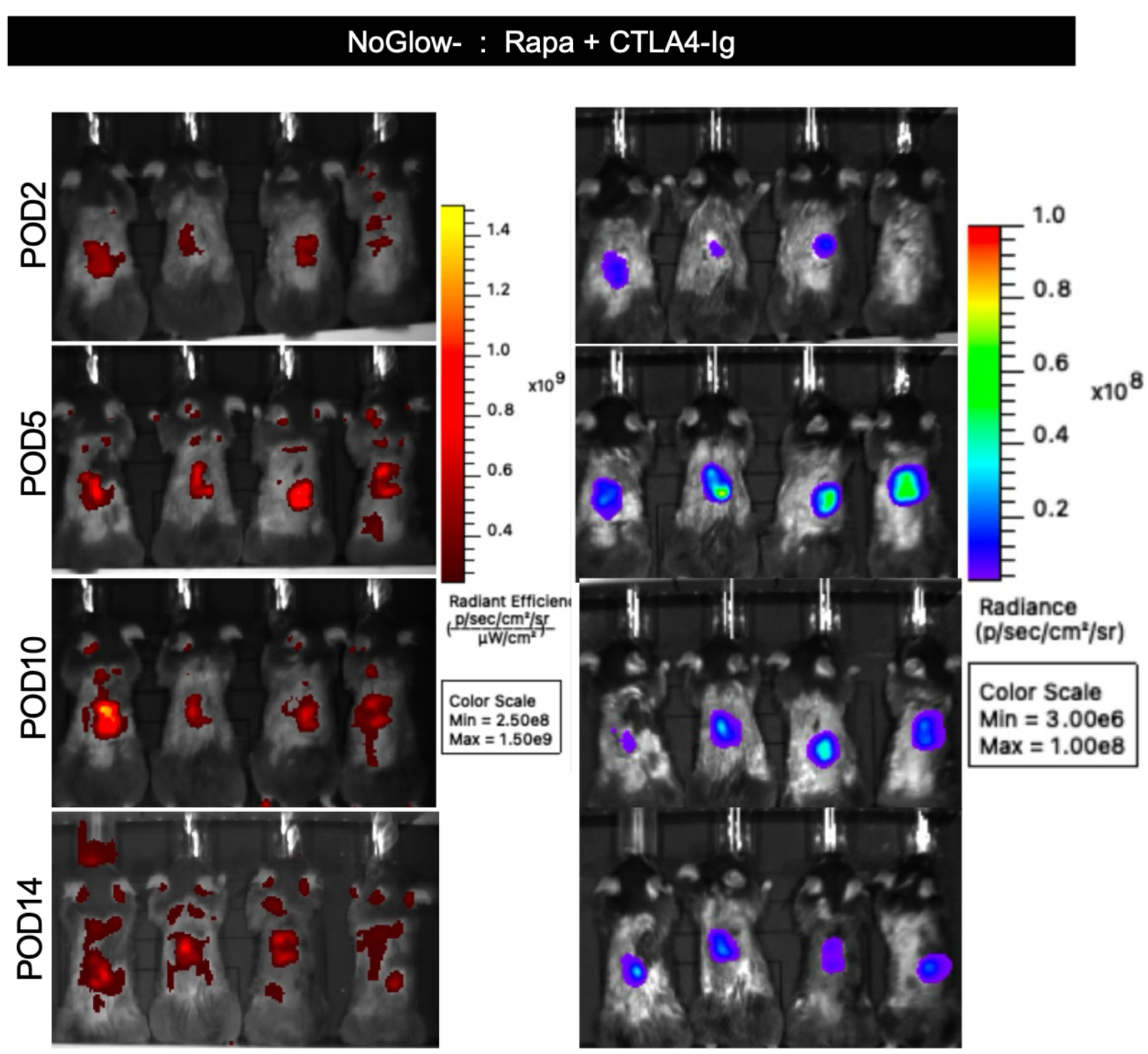
NoGlow- : Rapa + CTLA4-Ig
POD2
POD5
POD10
POD14
1.4
1.2
1.0
0.8
0.6
0.4
x10^9
Color Scale
Min = 2.50e8
Max = 1.50e9
1.0
0.8
0.6
0.4
0.2
x10^8
Radiance
(p/sec/cm²/sr)
Color Scale
Min = 3.00e6
Max = 1.00e8

Supplementary Figure 1. A-B. Photographs of transplanted CAG-eGFP-Luc skin grafts onto **(A)** NoGlow+ and untreated NoGlow- mice and **(B)** Belatacept and Rapamycin-treated NoGlow- mice over time. **C-D.** IVIS bioluminescent and fluorescent images of transplanted skin grafts onto **(C)** NoGlow+ and untreated NoGlow- mice and **(D)** Belatacept and Rapamycin-treated NoGlow- mice over time.

Supplementary Figure 2.

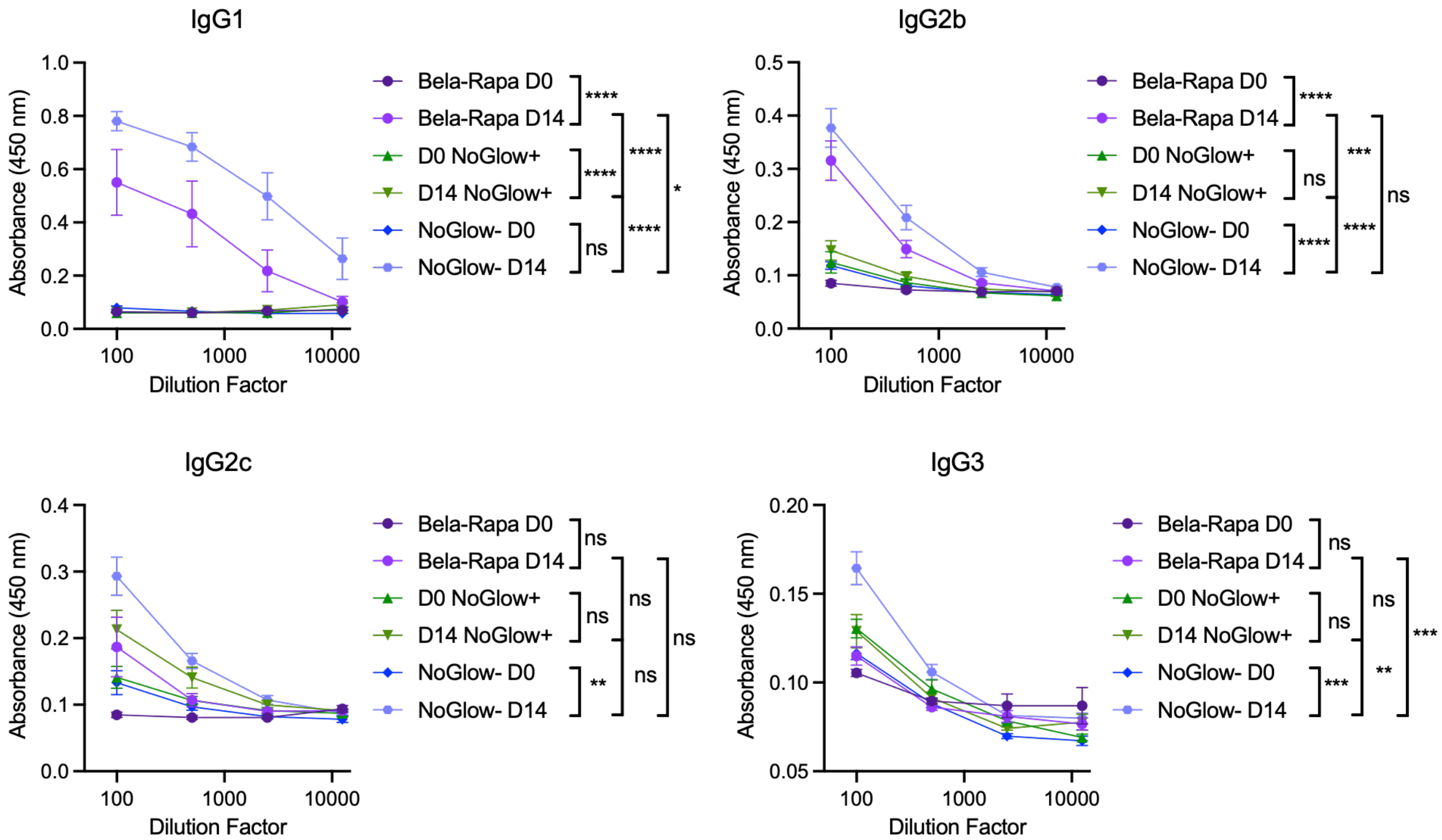

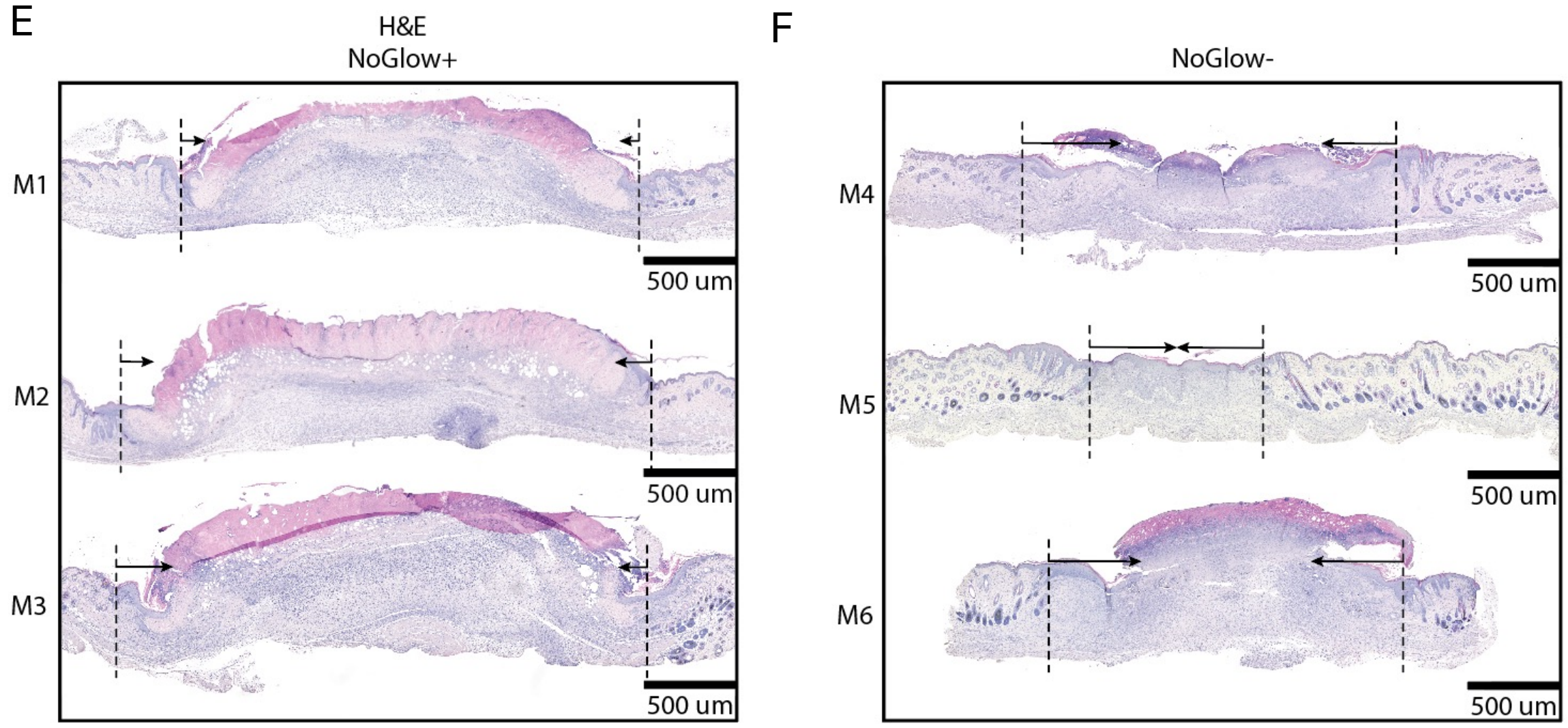
E
H&E
NoGlow+
M1
M2
M3
500 um
500 um
500 um
F
NoGlow-
M4
M5
M6
500 um
500 um
500 um

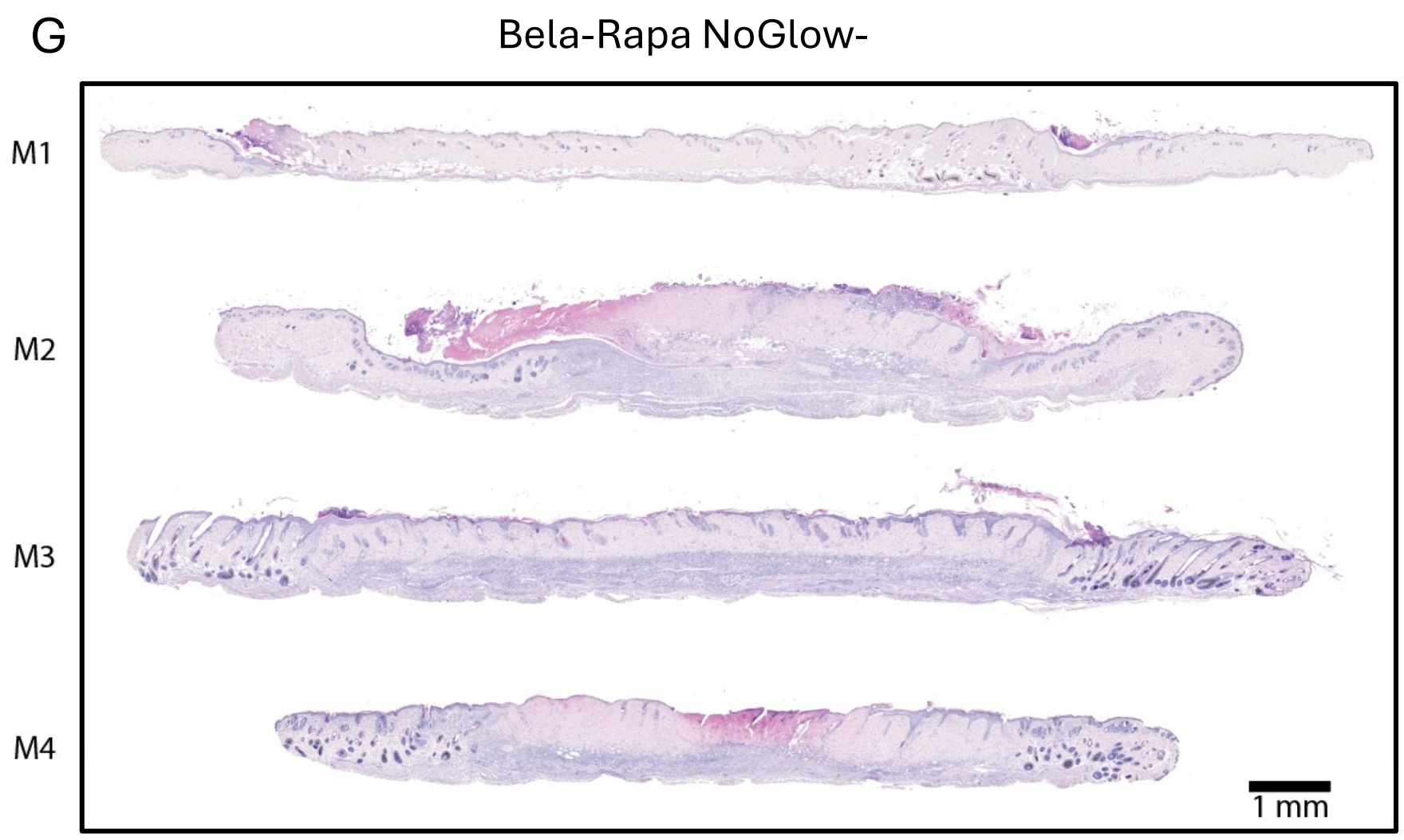
G
Bela-Rapa NoGlow-
M1
M2
M3
M4
1 mm

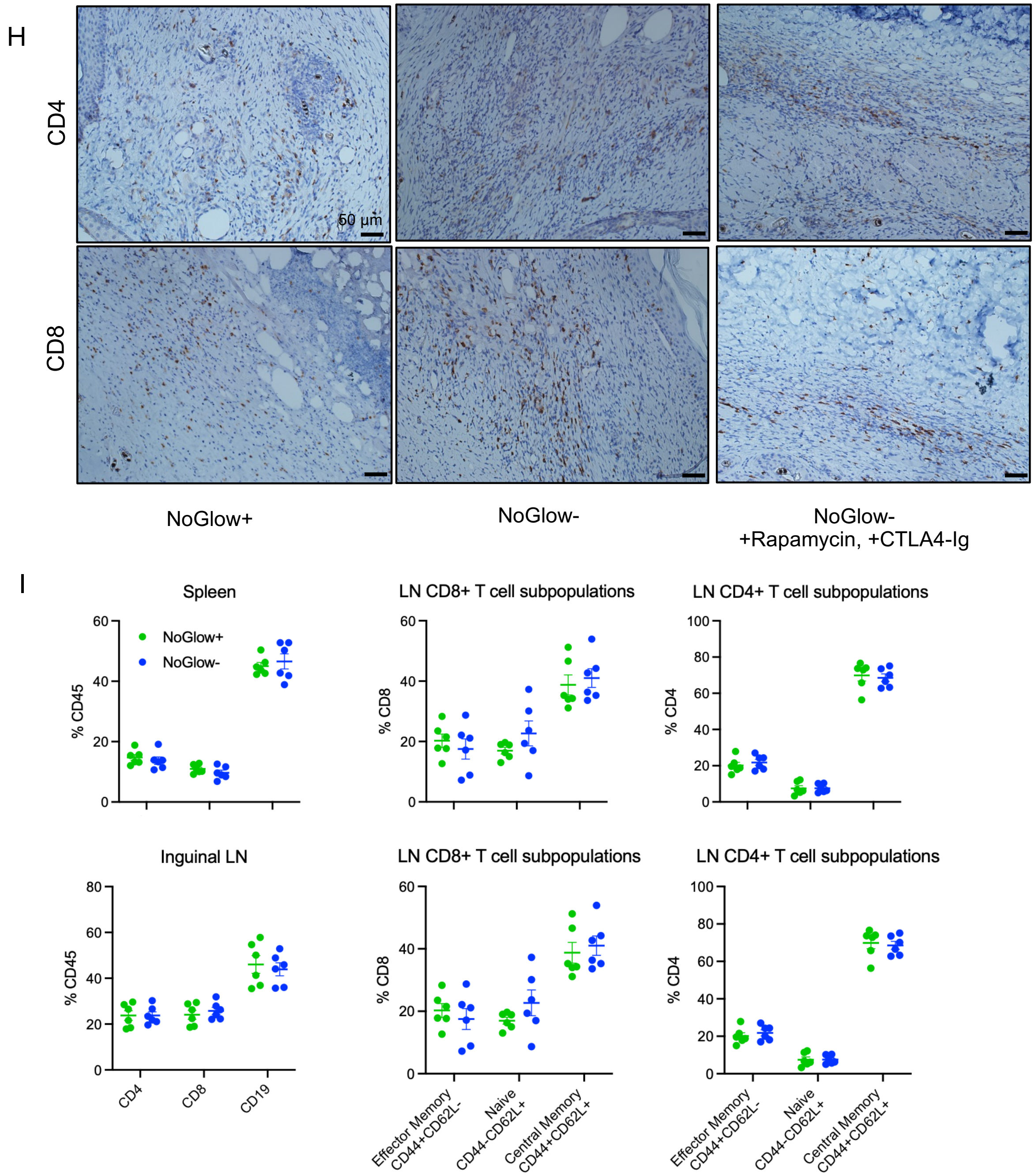

Supplementary Figure 2. A-D. anti-Luciferase IgG subclass ELISA data of **(A)** IgG1, **(B)** IgG2b, **(C)** IgG2c, **(D)** IgG3. **E-G.** Hematoxylin and Eosin (H&E) stained sections of **(E)** NoGlow+, **(F)** untreated NoGlow-, and **(G)** Belatacept-Rapamycin treated NoGlow- mice at endpoint. **H.** Representative images of graft-infiltrating CD4+ and CD8+ T cells in NoGlow+, NoGlow-, and NoGlow- + Rapamycin, CTLA4-Ig recipient mice. **I.** Frequencies of CD4+ and CD8+ T cells and CD19+ B cells within $CD45^{+}$ immune cell populations in spleen and inguinal lymph nodes at endpoint. $CD4^{+}$ and $CD8^{+}$ T cells were further classified into effector memory ($CD44^{+}CD62L^{-}$), naïve ($CD44^{-}CD62L^{+}$), and central memory ($CD44^{+}CD62L^{+}$) subsets. Data are presented as mean ± SEM. ELISA data (A-D) at 1:100 dilution were analyzed using one-way ANOVA with Holm-Sidak's multiple comparisons test. * P<0.05, **P<0.01, ***P<0.001, ****P<0.0001.

Supplementary Figure 3.

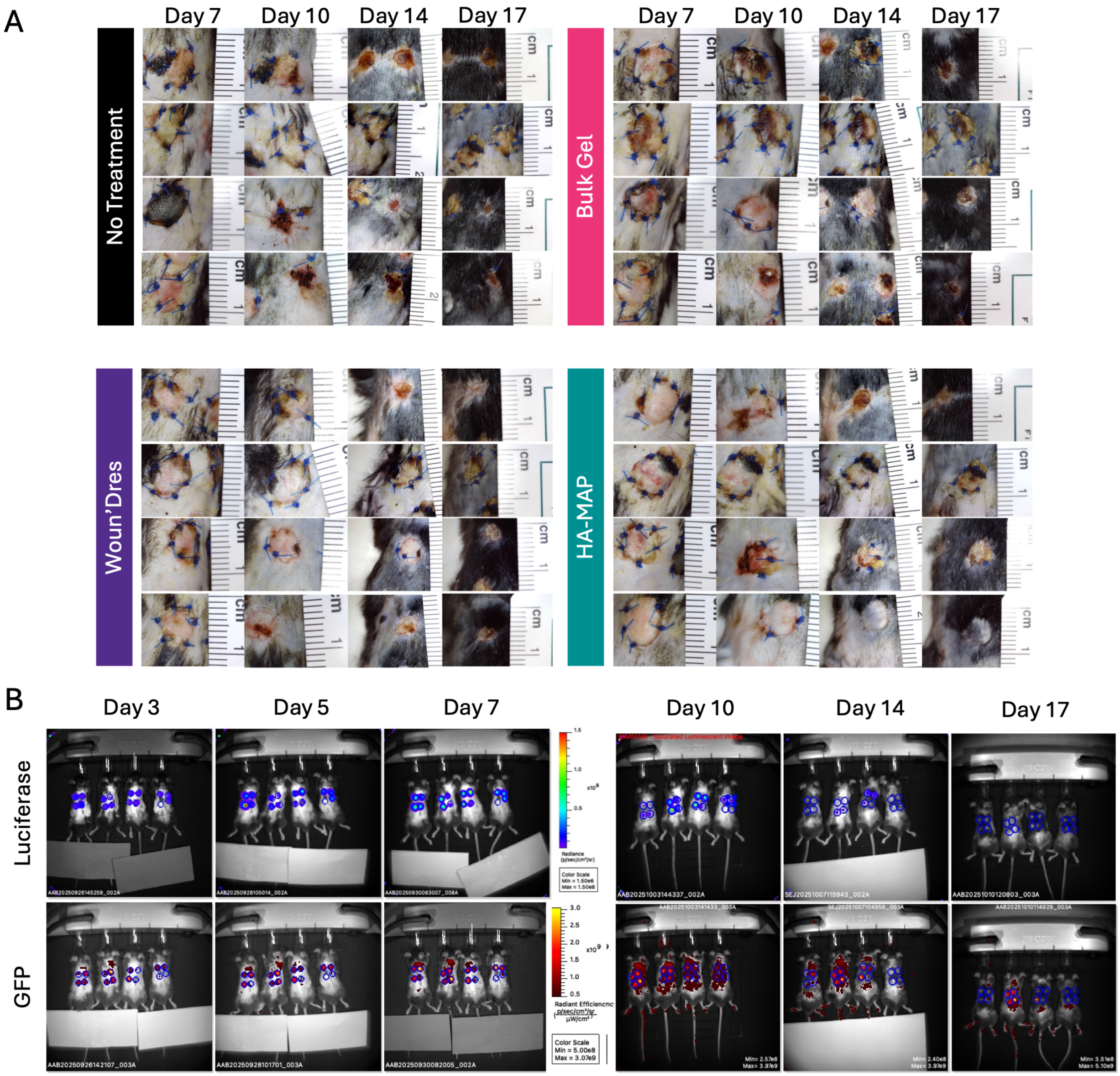

Supplementary Figure 3. A. Multiplex photographs of CAG-eGFP-Luc donor grafts onto NoGlow-recipients. **B.** Multiplex IVIS bioluminescence and fluorescence images.
